# UK B.1.1.7 (Alpha) variant exhibits increased respiratory replication and shedding in nonhuman primates

**DOI:** 10.1080/22221751.2021.1997074

**Published:** 2021-11-21

**Authors:** Kyle Rosenke, Friederike Feldmann, Atsushi Okumura, Frederick Hansen, Tsing-Lee Tang-Huau, Kimberly Meade-White, Benjamin Kaza, Julie Callison, Matthew C. Lewis, Brian J. Smith, Patrick W. Hanley, Jamie Lovaglio, Michael A. Jarvis, Carl Shaia, Heinz Feldmann

**Affiliations:** a Laboratory of Virology, Hamilton, MT, USA; b Rocky Mountain Veterinary Branch, Division of Intramural Research, National Institute of Allergy and Infectious Diseases, National Institutes of Health, Hamilton, MT, USA; c University of Plymouth, Plymouth, UK; d The Vaccine Group Ltd, Plymouth, UK

**Keywords:** SARS-CoV-2, variants of concern (VOC), African green monkey, transmission, virulence

## Abstract

The continuing emergence of SARS-CoV-2 variants calls for regular assessment to identify differences in viral replication, shedding and associated disease. In this study, we compared African green monkeys infected intranasally with either the UK B.1.1.7 (Alpha) variant or its contemporary D614G progenitor. Both variants caused mild respiratory disease with no significant differences in clinical presentation. Significantly higher levels of viral RNA and infectious virus were found in upper and lower respiratory tract samples and tissues from B.1.1.7 infected animals. Interestingly, D614G infected animals showed significantly higher levels of viral RNA and infectious virus in rectal swabs and gastrointestinal tissues. Our results indicate that B.1.1.7 infection in African green monkeys is associated with increased respiratory replication and shedding but no disease enhancement similar to human B.1.1.7 cases.

## Introduction

Severe acute respiratory syndrome coronavirus 2 (SARS-CoV-2) emerged in late 2019 as the causative agent of coronavirus disease 2019 (COVID-19). COVID-19 was declared a pandemic by the World Health Organization in March 2020 [1] and has now infected more than 224 million people with over 4.6 million deaths [2].

Enhanced sequence-based surveillance and epidemiological studies have led to the identification of multiple SARS-CoV-2 variants carrying distinct mutations that may impact transmissibility, disease severity and/or effectiveness of treatments and vaccines. The SARS-CoV-2 B.1.1.7 (Alpha) variant was first reported within the English county of Kent from the United Kingdom (UK) [3] and has since been classified as a “Variant of Concern” (VOC) associated with increased transmissibility and potentially increased disease severity but with minimal impact on the efficacy of monoclonal antibody treatment [4]. Several clinical reports supported the enhanced transmissibility associated with the B.1.1.7 VOC with up to a 90% increase in transmission compared to earlier variants [5–7]. However, the reported increase in mortality of the B.1.1.7 VOC [8–10] seen from earlier COVID case analysis has recently been questioned [11, 12]. Apart from clinical studies, experimental infections in animals – ideally in species closely related to humans such as nonhuman primates (NHPs) – is one way to assess viral shedding and disease severity of emerging SARS-CoV-2 variants.

The rhesus macaque model of SARS-CoV-2 infection was established early in the pandemic [13–15] and has been used to test SARS-CoV-2 therapeutics and vaccines [16–19]. Additional NHP species, such as cynomolgus macaques, baboons and marmosets have been investigated for their susceptibility to SARS-CoV-2 in an attempt to develop models exhibiting increased disease severity [20, 21]. None of these models result in severe disease, but susceptible NHP species exhibit oropharyngeal and nasal shedding and develop mild to moderate respiratory disease in the upper and lower respiratory tract. An African green monkey (AGM) model of SARS-CoV-2 infection has recently been developed, wherein animals show greater severity of disease [22–25], with intranasal infection of AGMs resulting in significant shedding and respiratory disease [23]. The AGM model may thereby represent a more natural NHP model for SARS-CoV-2 infection and disease.

In our current study, the AGM intranasal model of SARS-CoV-2 infection was used to assess differences between a contemporary progenitor SARS-CoV-2 variant (D614G), which was circulating in the summer of 2020, and the B.1.1.7 (Alpha) VOC that emerged from the D614G variant in the UK in late 2020. These variants were selected for direct comparison as they represent the two dominant SARS-CoV-2 variants circulating worldwide in late 2020 (D614G) and early 2021 (B.1.1.7) [26]. Herein, we report and discuss differences in organ tropism, replication kinetics and shedding between the two SARS-CoV-2 variants.

## Methods

*Biosafety and ethics*. All SARS-CoV-2 studies were approved by the Institutional Biosafety Committee (IBC) and performed in high biocontainment (BSL3/BSL4) at Rocky Mountain Laboratories (RML), NIAID, NIH. All sample processing in high biocontainment and sample removal followed IBC-approved Standard Operating Protocols (SOPs) [27]. All experiments involving AGMs were performed in strict accordance with approved Institutional Animal Care and Use Committee protocols and following recommendations from the Guide for the Care and Use of Laboratory Animals of the Office of Animal Welfare, National Institutes of Health and the Animal Welfare Act of the US Department of Agriculture, in an Association for Assessment and Accreditation of Laboratory Animal Care International (AAALAC)-accredited facility. AGMs were placed in a climate-controlled room with a fixed 12-hour light–dark cycle. Animals were singly housed in adjacent primate cages allowing social interactions and provided with commercial monkey chow, treats, and fruit twice daily with water *ad libitum*. Environmental enrichment was provided with a variety of human interaction, manipulanda, commercial toys, movies, and music. AGMs were monitored at least twice daily throughout the study.

*Virus and cells*. SARS-CoV-2 isolate SARS-CoV-2/human/USA/RML-7/2020 (MW127503.1), strain D614G (co-circulating progenitor), was obtained from a nasopharyngeal swab obtained on 19 July 2020. The original isolate was passaged twice on Vero E6 cells, deep sequencing of the viral stock showed it to be 100% identical to the deposited Genbank sequence and no contaminants were detected [28]. SARS-CoV-2 variant B.1.1.7 (hCoV-19/England/204820464/2020, EPI_ISL_683466) (Alpha) was obtained from Public Health England via BEI Resources (Manassas, VA, USA). The supplied passage 2 material was propagated once in Vero E6 cells. Deep sequencing confirmed the presence of three SNPs in this stock: nsp6 D156G (present in 14% of all reads), nsp6 L257F (18%) and nsp7 V11I (13%) [29].

Virus propagation was performed in Vero E6 cells in DMEM (Sigma-Aldrich, St Louis, MO, USA) supplemented with 2% fetal bovine serum, 1 mM L-glutamine, 50 U/ml penicillin and 50 μg/ml streptomycin (DMEM2). Vero E6 cells were maintained in DMEM supplemented with 10% fetal bovine serum, 1 mM L-glutamine, 50 U/ml penicillin and 50 μg/ml streptomycin (DMEM10). Mycoplasma testing of cell lines and viral stocks is performed regularly, and no mycoplasma was detected.

*Study design*. Twelve SARS-CoV-2 seronegative AGMs (3.8*–*6.7 kg) were randomly divided into 2 groups for infection with either the contemporary D614G progenitor variant (RML7) (*n *= 5; Table S1) or the B.1.1.7 (UK; Alpha) variant (*n *= 6; Table S1). Originally, 6 AGMs per group were assigned to each group but one animal in the D614G group had to be euthanized for an animal welfare concern reason prior to study start leaving the D614G group with only 5 animals. A Nasal Mucosal Atomization Device (Teleflex, MAD110) was used to deliver 10^6^ infectious particles (5 × 10^5^ per naris diluted in 500ul DMEM with no additives). Clinical examinations were performed on days 0, 1, 3, 5 and 7. Blood and serum were collected for hematology, blood chemistry, coagulation and virological analysis. Oropharyngeal, nasal and rectal swabs were collected at every examination for virological analysis. Bronchial cytology brushes were collected on days 3, 5 and 7 and bronchioalveolar lavage (BAL) samples were also collected on days 3 and 5 for virological analysis. Tissues were collected following euthanasia on day 7 for pathology and virological analysis. Studies were performed in successive weeks and different animal study groups to avoid contamination between studies, the D416G study was run first followed by the B.1.1.7 study.

*Virus titration*. Virus isolation was performed on tissues following homogenization in 1 mL DMEM using a TissueLyser (Qiagen, Germantown, MD, USA) and inoculating Vero E6 cells in a 96 well plate with 200 µL of 1:10 serial dilutions of the homogenate. One hour following inoculation of cells, the inoculum was removed and replaced with 200 µL DMEM. Virus isolation of blood and swab samples were performed in a similar manner. Samples were vortexed for 30 s before performing the 1:10 dilution series. The inoculum (200ul) was placed on cells and rocked for 1h. Infectious supernatant was removed and replaced with fresh DMEM. Seven days following inoculation, cytopathogenic effect was scored and the TCID_50_ was calculated using the Reed-Muench formula [30].

*Viral RNA detection*. qPCR was performed on RNA samples extracted from swabs or tissues using QiaAmp Viral RNA or RNeasy kits, respectively (Qiagen, Germantown, MD, USA). SARS-CoV-2 RNA was detected with one-step real-time RT–PCR assays designed to amplify viral RNA (vRNA; N gene) [31] or subgenomic RNA (sgRNA) only by amplifying a region of E gene to detect replicating virus [32]. Dilutions of RNA standards counted by droplet digital PCR were run in parallel and used to calculate viral RNA genome copies. A Rotor-Gene probe kit (Qiagen, Germantown, MD, USA) was used to run the PCRs according to the instructions of the manufacturer.

*Hematology, Serum Chemistry and Coagulation*. Hematology analysis was completed on a ProCyte DX (IDEXX Laboratories, Westbrook, ME, USA) and the following parameters were evaluated: red blood cells (RBC), hemoglobin (Hb), hematocrit (HCT), mean corpuscular volume (MCV), mean corpuscular hemoglobin (MCH), mean corpuscular hemoglobin concentration (MCHC), red cell distribution weight (RDW), platelets, mean platelet volume (MPV), white blood cells (WBC), neutrophil count (absolute number and percentage), lymphocyte count (absolute number and percentage), monocyte count (absolute number and percentage), eosinophil count (absolute number and percentage), and basophil count (absolute number and percentage). Serum chemistries were completed on a VetScan VS2 Chemistry Analyzer (Abaxis, Union City, CA, USA) and the following parameters were evaluated: glucose, blood urea nitrogen (BUN), creatinine, calcium, albumin, total protein, alanine aminotransferase (ALT), aspartate aminotransferase (AST), alkaline phosphatase (ALP), total bilirubin, globulin, sodium, potassium, chloride, and total carbon dioxide. Coagulation values were determined from citrated plasma utilizing a STart4 Hemostatis Analyzer and associated testing kits (Diagnostica Stago, Parsippany, NJ, USA).

*Serology*. Enzyme-linked immunosorbent assays were used to assess IgG and IgM antibody responses in each group. Plates were coated with Spike 1 or spike RBD antigen (The Native Antigen Company; 50 ng/well) in PBS for overnight adsorption at 4°C. Plates were washed in PBS/Tween (0.05) and wells blocked using 5% powdered milk in TBS/Tween (0.05%) for 1hr at RT. Serum samples were added at an initial 1:100 dilution followed by 1:4 dilutions up to 1:409,600 in duplicate and incubated 1 h at room temperature. Plates were washed and goat anti-rhesus IgG (H + L) HRP labelled secondary antibody (www.southernbiotech.com) or goat anti-monkey IgM HRP labelled secondary antibody (www.fitzgerald-fii.com) at 1:2000 dilution was added to all wells for 1hr at RT. After washing ABTS substrate (seracare) was added for 15 min before a 5% SDS solution was added to stop the reaction. Optical density (OD) values for each well were measured at 405 nm. Endpoint antibody titres were based on the last positive dilution of any particular dilution series. Positives were counted as any value above the average + 3 times standard deviation of the negative serum controls at each dilution.

*Cytokine analyses*. Concentrations of cytokines and chemokines present in the serum and BALs collected from SARS-CoV-2 infected AGMs were quantified using a multiplex bead-based assay (1:4 dilution) – the LEGENDPlex Non-Human Primate Cytokine/Chemokines 13-plex (BioLegend, San Diego, CA USA). Analytes detected by this panel were the following: IFN-γ, IL-1β, IL-6, IL-8, MCP-1, MIP-1α, MIP-1β, MIG, TNF-α, I-TAC, RANTES, IP-10, and Eotaxin. Samples were diluted 1:4 in duplicate prior to processing according the manufacturer’s instructions. Samples were read using the BD FACS Symphony instrument (BD Biosciences, San Jose, CA USA) and analyzed using LEGENDplexTM Data Analysis Software following data acquisition.

*Thoracic radiographs*. Ventro-dorsal and right/left lateral radiographs were taken on clinical exam days prior to any other procedures (e.g. bronchoalveolar lavage, nasal flush). Radiographs were evaluated and scored for the presence of pulmonary infiltrates by two board-certified clinical veterinarians according to a previously published standard scoring system [33]. Briefly, each lung lobe (upper left, middle left, lower left, upper right, middle right, lower right) was scored individually based on the following criteria: 0 = normal examination; 1 = mild interstitial pulmonary infiltrates; 2 = moderate interstitial pulmonary infiltrates, perhaps with partial cardiac border effacement and small areas of pulmonary consolidation (alveolar patterns and air bronchograms); and 3 = pulmonary consolidation as the primary lung pathology, seen as a progression from grade 2 lung pathology. At study completion, thoracic radiograph findings were reported as a single radiograph score for each animal on each exam day. To obtain this score, the scores assigned to each of the six lung lobes were added together and recorded as the radiograph score for each animal on each exam day. Scores range from 0 to 18 for each animal on each exam day.

*Histology and Immunohistochemistry*. Tissues were fixed in 10% neutral buffered formalin with two changes, for a minimum of 7 days according to an IBC-approved SOP. Tissues were processed with a Sakura VIP-6 Tissue Tek, on a 12-hour automated schedule, using a graded series of ethanol, xylene, and PureAffin. Embedded tissues were sectioned at 5 μm and dried overnight at 42°C prior to staining with hematoxylin and eosin. Specific staining was detected using SARS-CoV/SARS-CoV-2 nucleocapsid antibody (Sino Biological cat#40143-MM05) at a 1:1000 dilution. The tissues were processed for immunohistochemistry using the Discovery Ultra automated stainer (Ventana Medical Systems) with a ChromoMap DAB kit (Roche Tissue Diagnostics cat#760–159) (Roche Diagnostics Corp., Indianapolis, IN, USA).

*Statistical analyses*. Statistical analysis was performed in Prism 8 (GraphPad, San Diego, CA, USA). Area under the curve was used to analyze samples collected across multiple time points, no significant differences were found between the groups using these analyses. The multiple t-test function (95% CI) in prism was used to assess statistical significance between the two infection groups at individual timepoints.

## Results

**Figure 1.**
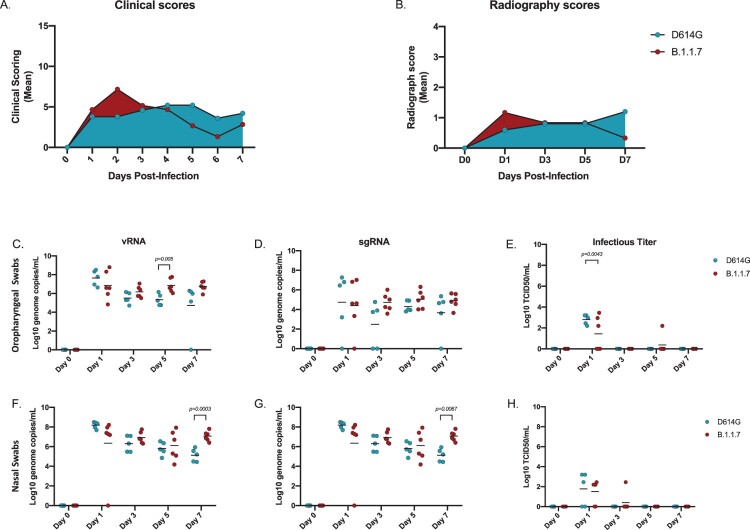
Clinical scoring, radiographs and viral loads in the URT. AGMs were infected with either the D614G or B.1.1.7 SARS-CoV-2 variant intranasally utilizing the Nasal Mucosal Atomization Device. (A) AGMs were scored daily for clinical signs of disease including changes in general appearance, respiration, food intake and feces as well as locomotion (B) Radiographs were taken on clinical exam days (0, 1, 3, 5, 7) and scored for pulmonary infiltrates. Swabs were taken on clinical exam days (0, 1, 3, 5 and 7) and used as a correlate for virus shedding. (vRNA) and (sgRNA) copies were determined by qRT-PCR. Infectious virus was titered on VeroE6 cells. (C-E) Viral loads in oropharyngeal swabs. Statistical significance was found at day 5 in vRNA (C, unpaired t-test, *p*-value <0.05) and at day 1 in infectious titers (E, unpaired t-test, *p*-value <0.05). (F-H) Viral loads in nasal swabs. Statistical significance was found at day 7 in vRNA (F, Multiple unpaired t-test, *p*-value <0.05) and at day 7 sgRNA (G, Multiple unpaired t-test, *p*-value <0.05). Multiple unpaired t-tests were used to compare the gRNA, sgRNA and infectious titers between groups (C-H) at individual time points.

*Infection with B.1.1.7 (Alpha) variant was not associated with a significant increase in disease severity in the AGM model*. Following intranasal infection with 1 × 10^6^ infectious particles of either the SARS-CoV-2 D614G (*n *= 5) or the B.1.1.7 variant (*n *= 6) (5 × 10^5^ per naris) using a nasal atomization device­­­, animals were monitored and scored daily for clinical signs of disease including changes in general appearance, respiration, food intake and fecal output and locomotion. Clinical signs were mild with both groups of AGMs displaying only minor changes in respiration and showing reduced appetite that negatively impacted volume of feces produced. Minor differences were observed between the two variant groups. B.1.1.7 infected animals had elevated scores early in infection peaking at 2 days post-infection (dpi), which subsequently returned toward baseline (Figure 1A). In contrast, scores for the D614G animals increased slowly peaking at 4 dpi and remained stable until euthanasia (Figure 1A, Table S1). Radiographs were taken at each examination and scored for pulmonary infiltrates, progression of which were similar to the clinical scores (Figure 1B, Table S2). B.1.1.7 infected AGMs scored minimally higher earlier and peaked at 1 dpi, whereas D614G infected animals scored higher later and peaked at 3 dpi. Overall, the minor changes in clinical and radiographic scores were not statistically significant between the two groups even though minor differences were noted in disease progression.

*B.1.1.7 (Alpha) variant replication/shedding from the upper respiratory tract was increased compared to D614G*. Oropharyngeal and nasal swabs were taken at each examination to assess virus replication in the upper respiratory tract (URT) and the potential for virus shedding. SARS-CoV-2 RNA was measured with qPCR assays targeting either vRNA (N assay) or sgRNA (E assay) (Figure 1C*–*H). vRNA in oropharyngeal swabs were significantly higher at 5 dpi in B.1.1.7 compared to D614G infected animals; this difference between variants was maintained but dropped below significance by 7 dpi (Figure 1C). There were no significant differences in sgRNA levels in oropharyngeal swabs at any time point, but levels consistently trended higher in the B.1.1.7 infection group starting at 3 dpi (Figure 1D). Although vRNA and sgRNA were detectable throughout the study, infectious virus was only isolated from oropharyngeal swabs at 1 dpi with significantly higher titers for the D614G infected animals (Figure 1E). Consistent with the oropharyngeal swabs, higher levels of vRNA and sgRNA were detected in nasal swabs collected from AGMs infected with B.1.1.7. By 7 dpi these animals were all shedding significantly more vRNA and sgRNA than those infected with D614G (Figure 1F, G). Infectious virus in the nasal swabs was recovered predominantly at 1 dpi and there was no difference between groups (Figure 1H).

**Figure 2.**
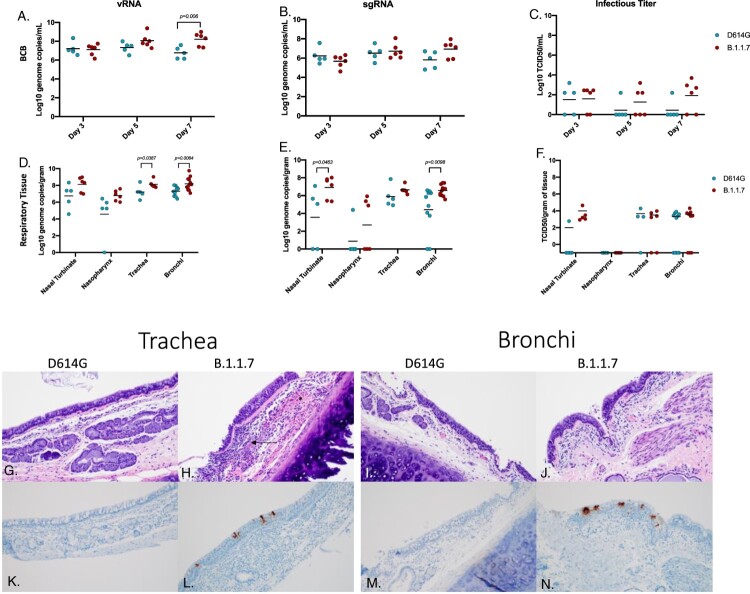
Virus load, pathology and virus antigen in the respiratory tract. AGMs were infected with either the D614G or B.1.1.7 SARS-CoV-2 variant intranasally utilizing the Nasal Mucosal Atomization Device. Bronchial cytology brush (BCB) samples were collected on days 3, 5 and 7 post-infection and samples were analyzed for vRNA, sgRNA and infectious virus. (A-C) A significant difference in vRNA collected in the BCBs was detected 7 days-post-infection (A, multiple unpaired t-test, *p*-value <0.05). Animals were euthanized on day 7 post-infection and respiratory tissues were collected for analyses for vRNA, sgRNA and infectious virus (D-F). vRNA was significantly different in the trachea and bronchi (D, multiple unpaired t-test, *p*-value <0.05). sgRNA differed significantly in the nasal turbinate and bronchi (E, multiple unpaired t-test, *p*-values <0.05). Infectious virus did not differ significantly in any tissue Infectious virus did not differ significantly in any tissue (F, multiple unpaired t-test were used to compare tissues between groups). Pathology and immunoreactivity in the trachea and bronchi (G-N). Normal trachea found in the D614G (G) vs. trachea with cellular infiltrates, hemorrhage (*) and fibrin (arrow) found in the submucosa in the B.1.1.7 (H). Immunoreactivity in the trachea of D614G vs B.1.1.7. (K, L, respectively) Normal bronchi found in the D614G (I) vs bronchi with inflammation and cellular infiltrates found in the B.1.1.7. (J) Immunoreactivity in bronchi of the D614G vs B.1.1.7 (M,N) (H&E and IHC magnifications (G_N) = 100x).

*B.1.1.7 (Alpha) variant replication in the respiratory tract was increased compared to D614G*. Samples to assess viral replication kinetics in the lower respiratory tract (LRT) were collected with broncho cytology brushes (BCB) at 3, 5 and 7 dpi and bronchoalveolar lavage (BAL) at 3 and 5 dpi. vRNA and sgRNA levels were consistent between both sampling methods (∼10^5^-10^7^ genome copies/ml) at each day sampled (Figure 2A–C; Fig S1). The vRNA levels were consistently higher in AGMs infected with B.1.1.7 and significantly increased at the final time points of BCB (7 dpi) (Figure 2A) and BAL (5 dpi) sampling (Fig S1). Although not significantly different at any time point, sgRNA was higher in the B.1.1.7 AGMs at 7 dpi in the BCB and in BAL at the final day of collection (5 dpi) (Figure 1B; Fig S1). Higher levels of infectious virus were also isolated from animals infected with B.1.1.7, particularly in BCB samples (Figure 2C; Fig. S1).

Post-mortem tissues were collected at 7 dpi for virological analysis and pathology. Respiratory tissues including nasal turbinate, nasopharynx, trachea and left and right bronchi and a section from each lung lobe were examined for viral RNA and infectious virus. vRNA and sgRNA were higher in all respiratory tissues in the B.1.1.7 infected animals and were significantly higher in the trachea and bronchi (Figure 2D,E). Although not statistically significant, AGMs infected with B.1.1.7 had higher levels of infectious virus in nasal turbinates, trachea and bronchi (Figure 2F). Consistent with higher levels of shedding in the nasal swabs, 5 out of the 6 animals (83%) infected with B.1.1.7 variant had SARS-CoV-2 immunoreactivity in the nasal epithelium compared to only 1 of 5 (20%) infected with the D614G (Fig S2). Similarly, in the trachea 5 out 6 animals (83%) infected with B.1.1.7 developed both inflammation and immunoreactivity with again only 1 of 5 animals (20%) infected with the D614G variant having any similar observable lesions or immunoreactivity (Figure 2G,H,K,L). Lesions were found in 8 of 10 bronchi (80%) from B.1.1.7 infected AGMs compared to only 2 of 8 (25%) of D614G animals, with lesions corresponding to SARS-CoV-2 immunoreactivity by immunohistochemistry (IHC) (Figure 2I,J,M,N).

**Figure 3.**
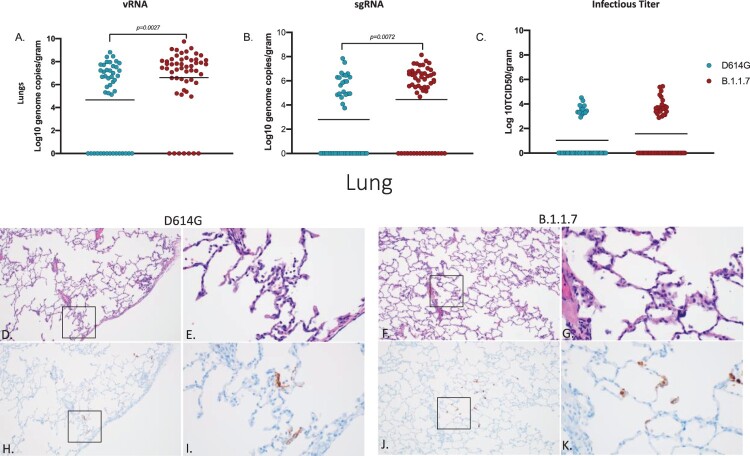
Virus load, pathology and immunoreactivity in the lungs. Animals were euthanized on day 7 post-infection and a section from each lung lobe was collected and analyzed for vRNA, sgRNA and infectious virus. Results of each assay were combined to look at the lungs in total (A-C). Significant differences were detected in lungs in both vRNA and sgRNA (D, multiple unpaired t-test, *p*-value <0.05 and E, multiple unpaired t-test, *p*-value <0.05) but not in infectious virus (C). Multiple unpaired t-tests were used to compare the two groups for statistical significance. Pathology and immunoreactivity in the lungs (D-K). Minimally thickened and inflamed alveolar septa with multifocal pneumocyte immunoreactivity in the D614G and B.1.1.7 samples (H&E (D,F) and IHC (H,J) = 100x; H&E (E,G) and IHC I,K) = 400x.).

vRNA and sgRNA were significantly higher in the lungs of B.1.1.7 – compared to D614G-infected animals (Figure 3A, B); however, elevated levels of infectious virus in B.1.1.7 animals remained just below statistical significance (Figure 3C). Lung lesions for both groups were minor but consistent with SARS-CoV-2 pneumonia and included thickening and inflammation of alveolar septa and the presence of fibrin (Figure 3D–G). SARS-CoV-2 immunoreactivity by IHC was also limited and not directly associated with foci of inflammation (Figure 3H–K).

**Figure 4.**
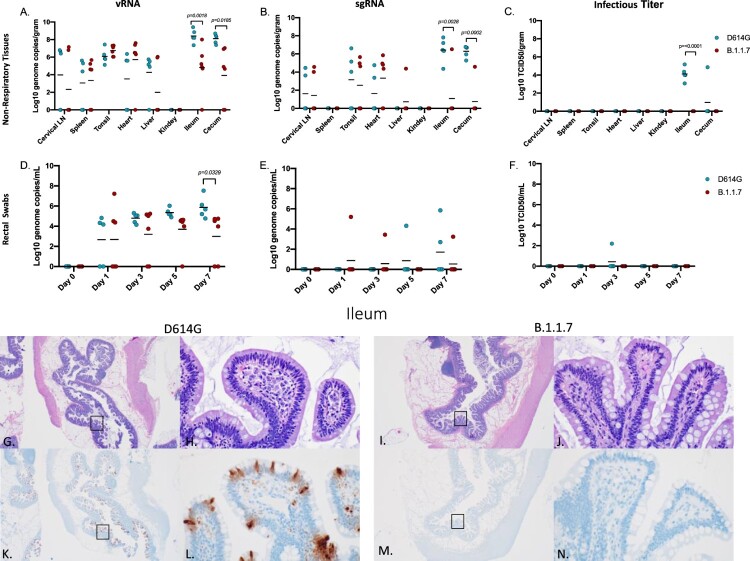
Viral loads in GIT and selected organs, GIT pathology and GIT immunoreactivity. AGMs were euthanized 7 days post-infection and tissues were collected to determine viral loads, pathology and immunoreactivity (A-C). vRNA and sgRNA was significantly different in the ileum and cecum (A, multiple unpaired t-test, *p*-value <0.05 and B, multiple unpaired t-test, *p*-value <0.05). Infectious virus was significantly different in the ileum (C, *p*-value <0.05), no other tissues were significantly different. (D-F) Viral loads in rectal swabs. Statistical significance was found at day 7 in vRNA (D, multiple unpaired t-test, *p*-value <0.05). Statistical significance was determined by multiple unpaired t-tests between the two groups. Pathology and immunoreactivity in the ileum (G-N). Normal mucosa with multifocal mucosal immunoreactivity in the D614G challenged ileum (G,H,K,L) (HE G, IHC K, 20x; HE H, IHC L, 400x). Normal mucosa and no immunoreactivity in the B.1.1.7 challenged ileum (I,J,M,N) (H&E (G,I) and IHC (K,M) = 20x; H&E (H,J) and IHC (L,N) - 400x).

*D614G variant replication in the gastrointestinal tract was increased compared to B.1.1.7.* vRNA and gRNA were detected in cervical lymph nodes, tonsil, heart, liver, spleen, ileum and cecum in both groups (Figure 4A). With the exception of one liver sample, sgRNA was not detected in liver, spleen nor kidneys (Figure 4B). Notably and in marked contrast to respiratory tissues, AGMs infected with D614G had significantly more vRNA and sgRNA in the ileum and cecum than B.1.1.7 infected AGMs (Figure 4A, B). Levels of vRNA and sgRNA corresponded to infectious virus with only D614G animals having detectable infectious SARS-CoV-2 in these two gastrointestinal tract (GIT)-derived tissues (Figure 4C). Similarly, vRNA in rectal swabs peaked and was significantly higher at 7 dpi in D614G infected animals (Figure 4D). sgRNA was recovered intermittently across the study (Figure 4E), but infectious virus was recovered from rectal swabs of only one (20%) D614G animal (Figure 4F). Viral replication in the ileum was associated with inflammation in all D614G infected AGMs and corresponded with detectable viral antigen by IHC (Figure 4G,H,K,L), while AGMs infected with the B.1.1.7 had no observable inflammation or viral antigen (Figure 4I,J,L,M). In D614G infected animals, only one AGM (20%) presented with inflammation and a small amount of associated viral antigen in the cecum. No infectious virus was isolated from any of the other non-respiratory tissue in either group and pathology was unremarkable in these tissues.

*Infection with neither variant was associated with marked changes in hematology, blood chemistry and coagulation, nor a broad systemic or local respiratory cytokine response*. Blood and serum samples were collected for hematology, blood chemistry, coagulation assays, cytokine analysis and serology at every clinical examination. No differences were found in the hematology (Fig S3A-L), blood chemistry (Fig S3M-T) or coagulation assays (Fig S4) between the D614G and B.1.1.7 infected AGMs. All animals showed an equivocal IgM titer at a dilution of 1:100 at 7 dpi with no IgG antibodies detectable (data not shown). Of the cytokines examined (Fig. S5), IL6 was the only pro-inflammatory cytokine that was significantly different between the groups, with IL6 being elevated in the D614G group at 3 and 5 dpi compared to B.1.1.7 infected animals (Fig S5A). Levels of T cell chemo-attractants IP-10 (CXCL 10) (Fig S5B) and I-Tac (CXCL 11) (Fig S5C) were also increased at 1 dpi in the D614G group but were not sustained throughout the study. Similarly, cytokine levels were examined in BAL samples collected at 3 and 5 dpi to determine local LRT responses. No significant differences were detected in the BAL samples at either time point between groups (Fig. S6).

## Discussion

The regular emergence of SARS-CoV-2 variants represents a constant public health challenge with the COVID-19 pandemic. Although epidemiological and clinical data can give insight into characteristics of a new variant, this data can be limited initially and may be biased by many factors. Animal models provide the ability to directly compare biological and clinical characteristics of multiple SARS-CoV2 variants in a study with limited variables providing invaluable data otherwise unattainable.

In the present study, we have used the AGM intranasal infection model to compare the B.1.1.7 (Alpha) VOC, a variant that emerged in the UK in September of 2020 and then quickly spread throughout the world [5, 7], with a contemporary D614G progenitor variant, in terms of virus replication, shedding and disease severity. A nasal atomization device was used to infect NHPs to most closely mimic natural infection in humans. Following infection with either variant, animals in both groups exhibited minor differences in disease progression but overall disease signs were similar with mild respiratory disease for both B.1.1.7 and D614G variants (Figure 1A,B; Table S1 & S2). Increased disease severity was initially reported for human B.1.1.7 cases [8-10], but more recent studies have contradicted these earlier claims [11, 12]. The outcome of our study using a NHP surrogate model supports findings from these more recent studies indicating that B.1.1.7 (Alpha) VOC is not associated with increased disease severity.

Although no animals in this study developed severe disease, our analysis reveal differences between the two variants in terms of their replication within the respiratory system. SARS-CoV-2 RNA and infectious virus in the LRT tissues were more prevalent in the animals infected with B.1.1.7 compared to D614G, especially at later timepoints suggesting the development of a stronger respiratory component associated with the emerging VOC (Figures 2 and 3). Consistent with higher levels of viral replication in the URT, shedding of SARS-CoV-2 RNA in both the nose and oropharyngeal cavity was also higher in B.1.1.7 infected animals suggestive of potentially enhanced transmissibility of the B.1.1.7 (Alpha) over the D614G progenitor variant (Figure 1). This supports reports from human infection data showing that the B.1.1.7 VOC is more transmissible than earlier variants [6, 7, 34].

The pathology associated with infections differed between the two variants. B.1.1.7 replicated at higher levels in the respiratory tract resulting in lesions that were both more numerous and severe than seen for D614G infected animals. In contrast, D614G replicated at higher levels in the GIT and the associated pathology seen in these animals correlated with this difference in GIT replication. This finding was supported by higher levels of SARS-CoV-2 RNA in rectal swabs (Figure 4). This may indicate that D614G is more suited to replication in the GIT than other variants which is in line with clinical studies conducted in early to mid-2020 that reported GIT symptoms in approximately 15-20% of COVID-19 patients [35]. The difference observed in these studies may also indicate that genetic alterations in B.1.1.7 may have not merely resulted in a general increased rate of replication but may have also altered organ tropism. Clinical studies concerning B.1.1.7 have to date been mainly focused on respiratory symptoms [8–11]. However, it remains to be seen whether the observed changes in tissue tropism and replication detected here in the AGM model will correspond to a drop in reported GIT symptoms in those infected with the B.1.1.7 VOC compared to infections with contemporary SARS-CoV-2. It is also possible that AGMs may be more prone to GIT infections and an earlier SARS-CoV-2 study with AGMs using the nCoV-WA1-2020 isolate suggests this could be true. That study had a single animal that exhibited infection in the GIT up to 10 dpi but the remaining animals did not [36]. Future studies should address potential differences in organ tropism associated with SARS-CoV-2 variants.

Several SARS-CoV-2 models have been described utilizing different NHP species such as macaques [13–16], baboons [20], marmosets [20, 21] and AGMs [22, 23]. These studies used different infectious doses and routes of infection resulting in variation and inconsistency regarding clinical, virological and pathological parameters measured. NHP species may also differ in their susceptibility to SARS-CoV-2 which further complicates the comparisons between studies using different species. Furthermore, the challenge variant stocks used for infection may have genetic variability acquired in nature or by passaging in tissues culture. All these factors affect study outcomes and make comparative interpretations difficult, a limitation that is associated with all of the NHP models. A recent study examining SARS-CoV-2 variants in the rhesus macaque model showed no difference in viral replication nor disease between the D614G and B.1.1.7 variants [29]. Notably, this study used double the infectious dose via intranasal challenge using the same atomizing device, but also added an intratracheal inoculation route [29]. Although AGMs and rhesus macaques share similar ACE-2/RBD binding affinities [37], we cannot rule out the different NHP species as a potential factor in the outcome of infection. The marginally higher dose and additional route of infection may have been additional factors affecting infection outcomes. In our study we purposely selected the AGM species for the intranasal challenge model, chose intranasal challenge to mimic a likely natural exposure route, used a single route of infection for the ease of analyzing viral spread, and a high dose of infection for maximum disease.

In conclusion, our results from the intranasal AGM COVID-19 surrogate model support the most recent data from B.1.1.7 in humans, providing direct empirical data for increased replication in the respiratory tissue, but with no enhancement of disease. Although the lack of clear statistical significance for some of the parameters may be regarded as possible limitation of the study, the use of these multiple parameters to independently verify one another addresses this concern. One way to obtain statistical significance more uniformly is to increase NHP numbers something that is ethically sensitive and difficult given the current issues with NHP availability. NHPs remain a surrogate model for humans and results presented herein provide direct experimental evidence that support recent clinical observations, which indicate that the B.1.1.7 VOC has characteristics of increased replication in respiratory tissues with enhanced shedding from the URT resulting in potentially enhanced transmission. A further notable and interesting observation of differences between these two variants in terms of a potential distinct viral tissue/organ tropism warrants further attention as such changes would have public health implications in terms of transmission and disease manifestation.

## Data and materials availability

All data are available in the main text or the supplementary materials. Additional information can be requested through the corresponding author.

## Supplementary Material

Clean_copy_of_supplementary_materials.docx
