# Supplementary material for: UK B.1.1.7 (Alpha) variant exhibits increased respiratory replication and shedding in nonhuman primates
Source: Emerg Microbes Infect. 2021 Nov 21;10(1):2173–82. doi: 10.1080/22221751.2021.1997074 (PMC8635622; doi:10.1080/22221751.2021.1997074)
Supplement: Clean_copy_of_supplementary_materials.docx [file TEMI_A_1997074_SM2028.docx]

**SUPPLEMENTARY FIGURES**

**Figure S1: Viral loads in the lower respiratory tract (BAL).**

AGMs were infected with either the D614G or B.1.1.7 SARS-CoV-2 variant intranasally utilizing a Nasal Mucosal Atomization Device. Bronchioalveolar lavage (BAL) samples were collected on 3 and 5 days post-infection (dpi) and measured for gRNA, sgRNA and infectious titers. (A-C) A significant difference in gRNA collected in BAL samples was detected on 5dpi (*p-value <0.05), no other significant differences were detected. Multiple t-tests were used to compare groups.

**Figure S2: Pathology in the nasal turbinates.** AGMs were infected with either the D614G or B.1.1.7 SARS-CoV-2 variant intranasally utilizing the Nasal Mucosal Atomization Device. Pathology and immunoreactivity in the nasal epithelium (A-D). (A, C) Normal epithelium found in the D614G vs. epithelium with immunoreactivity in the nasal turbinates of B.1.1.7. infected animals (B, D) (HE A, B; IHC C, D 400x).

**Figure S3: Hematology and blood chemistry following infection.** Whole blood and serum samples were collected at each exam time point (0, 1, 3, 5 and 7dpi) for hematology (A-L) and blood chemistry analyses (M-T). No significant changes were found in hematology (A-L), nor in blood chemistry (M-T).

**Figure S4: Coagulation assays following infection.** Plasma samples were collected at each clinical time point (0, 1, 3, 5 and 7dpi) to evaluate coagulation parameters between infected animals (A-D). No significant changes were found in PT (A), APTT (B), fibrinogen (C) or thrombin (D).

**Figure S5: Systemic cytokine analyses following infection.** Serum was collected on 0, 1, 3, 5dpi for cytokine analyses. Three notable changes were detected. Levels of IL-6 were significantly different at 3dpi between the two groups (*p-value 0.05) (A). Differences at 1dpi were noted in both IP-10 (B) and I-TAC (C) but were not significant. Samples were analyzed by 2-way ANOVA to determine significance.

**Figure S6: Localized cytokine analyses of BALs following infection.** BALs were collected on 3 and 5dpi for cytokine analyses. No notable changes were detected between groups. Samples were analyzed by multiple tests to determine statistical significance.

**Table S1: Clinical scoring and necropsy notes of infected animals.** AGMs were scored daily for clinical signs of disease including changes in general appearance, respiration, food intake, fecal output as well as locomotion. Macroscopic scoring of organs was performed during necropsies (day 7 post-infection).

**Table S2: Radiographic scoring of lungs following infection.** Ventro-dorsal and right/left lateral radiographs were taken on clinical exam days prior to any other procedures (e.g. bronchoalveolar lavage, nasal flush). Radiographs were evaluated and scored for the presence of pulmonary infiltrates by two board-certified clinical veterinarians according to a standard scoring system (*1*). Briefly, each lung lobe (upper left, middle left, lower left, upper right, middle right, lower right) was scored individually based on the following criteria: 0 = normal examination; 1 = mild interstitial pulmonary infiltrates; 2 = moderate interstitial pulmonary infiltrates, perhaps with partial cardiac border effacement and small areas of pulmonary consolidation (alveolar patterns and air bronchograms); and 3 = pulmonary consolidation as the primary lung pathology, seen as a progression from grade 2 lung pathology. At study completion, thoracic radiograph findings were reported as a single radiograph score for each animal on each exam day. To obtain this score, the scores assigned to each of the six lung lobes were added together and recorded as the radiograph score for each animal on each exam day. Scores can range from 0 to 18 for each animal on each exam day.

**References**

1. D. L. Brining *et al.*, Thoracic radiography as a refinement methodology for the study of H1N1 influenza in cynomologus macaques (Macaca fascicularis). *Comp Med* **60**, 389-395 (2010).
